# Correction: Harper et al. Alberta Childhood Cancer Survivorship Research Program. *Cancers* 2023, *15*, 3932

**DOI:** 10.3390/cancers16244160

**Published:** 2024-12-13

**Authors:** Andrew Harper, Fiona Schulte, Gregory M. T. Guilcher, Tony H. Truong, Kathleen Reynolds, Maria Spavor, Natalie Logie, Joon Lee, Miranda M. Fidler-Benaoudia

**Affiliations:** 1 Department of Cancer Epidemiology and Prevention Research, Cancer Care Alberta, Alberta Health Services, Calgary, AB T2S 3C3, Canada; andrew.harper@ahs.ca; 2 Department of Oncology, Cumming School of Medicine, University of Calgary, Calgary, AB T2N 1N4, Canada; fsmschul@ucalgary.ca (F.S.); greg.guilcher@ahs.ca (G.M.T.G.); tony.truong@ahs.ca (T.H.T.); 3 Long Term Survivor’s Clinic, Alberta Children’s Hospital, Calgary, AB T2N 4N1, Canada; kathya.reynolds@ahs.ca; 4 Department of Pediatrics, Cumming School of Medicine, University of Calgary, Calgary, AB T2N 4N1, Canada; 5 Department of Medicine, Faculty of Family Medicine, University of Calgary, Calgary, AB T2N 4N1, Canada; 6 Department of Pediatrics, Faculty of Medicine and Dentistry, University of Alberta, Edmonton, AB T6G 2R7, Canada; maria.spavor@ahs.ca; 7 Division of Radiation Oncology, Tom Baker Cancer Centre, Alberta Health Services, Calgary, AB T2N 4N2, Canada; natalie.logie@ahs.ca; 8 Data Intelligence for Health Lab, Cumming School of Medicine, University of Calgary, Calgary, AB T2N 4N1, Canada; joonwu.lee@ucalgary.ca; 9 Department of Cardiac Sciences, Cumming School of Medicine, University of Calgary, Calgary, AB T2N 4N1, Canada; 10 Department of Community Health Sciences, Cumming School of Medicine, University of Calgary, Calgary, AB T2N 4N1, Canada

**Affiliation Correction**

In the published manuscript [1], there was an error regarding the affiliation 5. The wrong affiliation “Department of Supplementary Medicine, Faculty of Family Medicine, University of Calgary, Calgary, AB T2N 4N1, Canada” is now corrected to “Department of Medicine, Faculty of Family Medicine, University of Calgary, Calgary, AB T2N 4N1, Canada”.

**Text Correction**

There were errors in the original publication [1]. An error in data cleaning resulted in incorrect numbers being shown throughout the abstract and text of the manuscript.

Corrections have been made to the Simple Summary:

Treatments used to cure childhood cancer can have negative long-term impacts on physical health and well-being. Here, we present the Alberta Childhood Cancer Survivorship Research Program, its foundational cohort, and descriptive statistics of outcomes ascertained through data linkage. To this end, 2581 survivors of childhood cancer were included in the cohort, the majority of which were male, diagnosed between the ages of 0 and 4 years, with leukemia, central nervous system tumor, or lymphoma. By the study exit date, the median time since diagnosis was 5.6 years overall and 10.3 years for 5-year survivors. During the follow-up time, 94 subsequent cancers were diagnosed, 16,669 inpatient and 445,150 ambulatory/outpatient events occurred, 396,074 claims were reported, and 408 survivors died. The results from this research program seek to inform and improve clinical care and reduce cancer-related sequelae.

Corrections have been made to the Abstract:

Adverse outcomes after childhood cancer have been assessed in a range of settings, but most existing studies are historical and ascertain outcomes only after 5-year survival. Here, we describe the Alberta Childhood Cancer Survivorship Research Program and its foundational retrospective, population-based cohort of Albertan residents diagnosed with a first primary neoplasm between the ages of 0 and 17 years from 1 January 2001 to 31 December 2018. The cohort was established in collaboration with the Alberta Cancer Registry and Cancer in Young People in Canada program and has been linked to existing administrative health databases and patient-reported outcome questionnaires. The cohort comprised 2581 survivors of childhood cancer, 1385 (53.7%) of whom were 5-year survivors. Approximately 48% of the cohort was female, 46% of the cohort was diagnosed between 0 and 4 years of age, and the most frequent diagnoses were leukemias (25.3%), central nervous system tumors (24.2%), and lymphomas (14.9%). Detailed treatment information was available for 1745 survivors (67.6%), with manual abstraction ongoing for those with missing data. By the study exit date, the median time since diagnosis was 5.6 years overall and 10.3 years for 5-year survivors. During the follow-up time, 94 subsequent primary cancers were diagnosed, 16,669 inpatient and 445,150 ambulatory/outpatient events occurred, 396,074 claims were reported, and 408 survivors died. The results from this research program seek to inform and improve clinical care and reduce cancer-related sequelae via tertiary prevention strategies.

Corrections have been made to the Materials and Methods:

2.4.4. Practitioner Claims

The Practitioner Claims database contains information about the service utilization of physicians and their patients as well as payment information [32]. These data are collected through claims submitted under an Alberta Health Care Insurance Plan (AHCIP) [25]. Data are coded for billing purposes by physicians or their staff, with up to three diagnoses coded using the ICD, 9th Revision, Clinical Modification [36], and one procedure is coded using the Canadian Classification of Diagnostic, Therapeutic, and Surgical Procedures. For the purposes of this project, data were available from 1 January 2001 onwards.

Corrections have been made to the Results:

*3.1. Cohort Characteristics*

Table 1 and Figure 1 present the descriptive characteristics of the cohort overall and for 5-year survivors (i.e., children who survived at least five years from their date of cancer diagnosis). Overall, a total of 2581 children were diagnosed with a first primary neoplasm before the age of 18 years in Alberta, Canada between 1 January 2000 and 31 December 2018, 1385 (53.7%) of whom were 5-year survivors. Approximately 48% of the cohort was female, with approximately 36% of cases occurring in the Calgary zone and 32% of cases occurring in the Edmonton zone. Nearly 40% of the cohort was diagnosed between 0 and 4 years of age, and the most frequent diagnoses were leukemias, myeloproliferative diseases, and myelodysplastic diseases (25.3%), central nervous system (CNS) and miscellaneous intracranial and intraspinal neoplasms (24.2%), and lymphomas and reticuloendothelial neoplasms (14.9%). Descriptive characteristics were generally similar when restricted to 5-year survivors.

For individuals with detailed treatment information available (*n* = 1745; 67.6%), nearly all received radiotherapy, chemotherapy, and/or surgery. Chemotherapy only was the most common form of treatment, followed by chemotherapy and surgery, and chemotherapy, radiotherapy, and surgery. Detailed descriptive statistics for chemotherapy agents and radiotherapy sites are shown in Table 2 and Table S1. The most common chemotherapy agents were Vincristine and Cyclophosphamide, with a median dose of 3002.6 mg/m^2^ observed for the latter. In total, 525 individuals received radiotherapy, and doses were available for 513 (97.7%) individuals. The median dose was 2550 cGy, and the most frequently irradiated sites were the CNS (*n* = 266), abdomen (*n* = 86), and sites other than those specified (*n* = 85). Compared with the overall cohort, individuals for whom detailed treatment information was not available were more likely to be diagnosed with a central nervous system tumor (29.6% vs. 24.2%), be older at diagnosis (median 15.4 years vs. 7.6 years), and not be residing in Calgary (34.1% vs. 35.5%) or Edmonton (29.9% vs. 31.6%) zones (Table 3). Manual treatment abstraction is ongoing for all those individuals missing detailed treatment information.

By the study exit date (31 December 2018), the median attained age was 15.1 years (IQR: 8.7–20.8), and the median follow-up time from cancer diagnosis was 5.6 years (IQR: 1.9–10.9) (Table 1). As expected, the median attained age and follow-up time increased when restricted to 5-year survivors to 19.7 (IQR: 14.0–24.5) and 10.3 (IQR: 7.5–13.7), respectively.

*3.2. Late Effects*

At the time of publication, data linkages have been undertaken with all population-based registries and administrative health databases. Work is ongoing to abstract late effects’ data from the LTSQs, which were paper-based until 2020. The results are thus only presented for outcomes where data collection is complete.

Figure 2 shows a flowchart of all data linkages. SPNs and vital status information were ascertainable for the entire cohort (Table 4). A total of 94 SPNs were observed among 83 survivors of childhood cancer, 55 of which were observed after 5-year survivorship in 48 survivors. In terms of mortality, 408 (15.8%) of the survivors of childhood cancer included in the overall cohort died by the study exit date, though the proportion substantially decreased when restricted to 5-year survivors, at 2.7%. For the survivors of childhood cancer that were able to be linked to the administrative health databases (i.e., not missing a ULI (*n* = 1) and who were not dead or lost to follow-up before the beginning of each database), the following numbers were observed: 2254 (87.3%) appeared in DAD, with a total of 16,669 records discharged; 2533 (98.1%) appeared in NACRS, with 445,150 records ambulatory/outpatient events observed; and 2549 (98.8%) appeared in Practitioner Claims, equating to 396,074 records. The corresponding number of survivors and observed records for each database for 5-year survivors can be found in Table 4. It is worthwhile to note that some events from these databases may be related to care or side effects occurring during the treatment phase, particularly in regard to events before 5-year survivorship, and that not all healthcare utilization will be related to their past cancer diagnosis.

**Error in Figure/Table**

In the original publication, there were mistakes in the tables and figures as published. An error in data cleaning resulted in incorrect numbers being shown in all tables and figures included in the manuscript.

The corrected Table 1 appears below:

**Table 1:** Cohort characteristics, overall and among 5-year survivors.

Characteristic	Overall Number (%)	5-Year Survivors Number (%)
Total	2581 (100.0)	1385 (100.0)
Sex		
Male	1354 (52.5)	740 (53.4)
Female	1227 (47.5)	645 (46.6)
Time Since Diagnosis (years) ^1^		
0–<5	1196 (46.3)	0 (0.0)
5–<10	627 (24.3)	627 (45.3)
10–<14	522 (20.2)	522 (37.7)
≥15	236 (9.1)	236 (17.0)
Median (IQR)	5.6 (1.9–10.9)	10.3 (7.5–13.7)
ICCC-3 Diagnosis Category		
Leukemias, myeloproliferative diseases, and myelodysplastic diseases	654 (25.3)	364 (26.3)
Lymphomas and reticuloendothelial neoplasms	385 (14.9)	233 (16.8)
CNS and miscellaneous intracranial and intraspinal neoplasms	624 (24.2)	314 (22.7)
Neuroblastoma and other peripheral nervous cell tumors	161 (6.2)	81 (5.9)
Retinoblastoma	57 (2.2)	38 (2.7)
Renal tumors	109 (4.2)	69 (5.0)
Hepatic tumors	48 (1.9)	19 (1.4)
Malignant bone tumors	124 (4.8)	56 (4.0)
Soft tissue and other extraosseous sarcomas	148 (5.7)	68 (4.9)
Germ cell tumors, trophoblastic tumors, and neoplasms of gonads	109 (4.2)	61 (4.4)
Other malignant epithelial neoplasms and malignant melanomas	153 (5.9)	78 (5.6)
Other and unspecified malignant neoplasms	9 (0.4)	4 (0.3)
Year of Diagnosis		
2001–2005	620 (24.0)	502 (36.3)
2006–2010	676 (26.2)	549 (39.6)
2011–2015	786 (30.5)	334 (24.1)
2016–2018	499 (19.3)	0 (0.0)
Age at Diagnosis (years)		
0–4	1000 (38.7)	510 (36.8)
5–9	503 (19.5)	269 (19.4)
10–14	554 (21.5)	312 (22.5)
15–17	524 (20.3)	294 (21.2)
Median (IQR)	7.6 (2.9–14.1)	8.1 (3.2–14.4)
Attained Age (years) ^1^		
0–4	318 (12.3)	0 (0.0)
5–9	451 (17.5)	134 (9.7)
10–14	510 (19.8)	279 (20.1)
15–19	582 (22.6)	302 (21.8)
20–24	404 (15.7)	354 (25.6)
25–29	226 (8.8)	226 (16.3)
30–34	89 (3.5)	89 (6.4)
≥35	1 (<0.1)	1 (0.1)
Median (IQR)	15.1 (8.7–20.8)	19.7 (14.0–24.5)
CYP-C Treatment		
No treatment	70 (2.7)	19 (1.4)
CT only	588 (22.8)	317 (22.9)
RT only	10 (0.4)	2 (0.1)
Surgery only	215 (8.3)	127 (9.2)
CT + RT	154 (6.0)	81 (5.9)
CT + Surgery	347 (13.4)	178 (12.9)
RT + Surgery	71 (2.8)	39 (2.8)
CT + RT + Surgery	290 (11.2)	140 (10.1)
Patients with missing treatment information	836 (32.4)	482 (34.8)
Zone of Diagnosis		
South	164 (6.4)	89 (6.4)
Calgary	915 (35.5)	471 (34.0)
Central	343 (13.3)	202 (14.6)
Edmonton	815 (31.6)	440 (31.8)
North	343 (13.3)	182 (13.1)
Alberta, zone unknown	1 (<0.1)	1 (0.1)

^1^ Censored to date of death, last known date (i.e., loss to follow-up), or end of study (i.e., 31 December 2018), whichever occurs first.

The corrected Figure 1 appears below:

**Figure 1:**
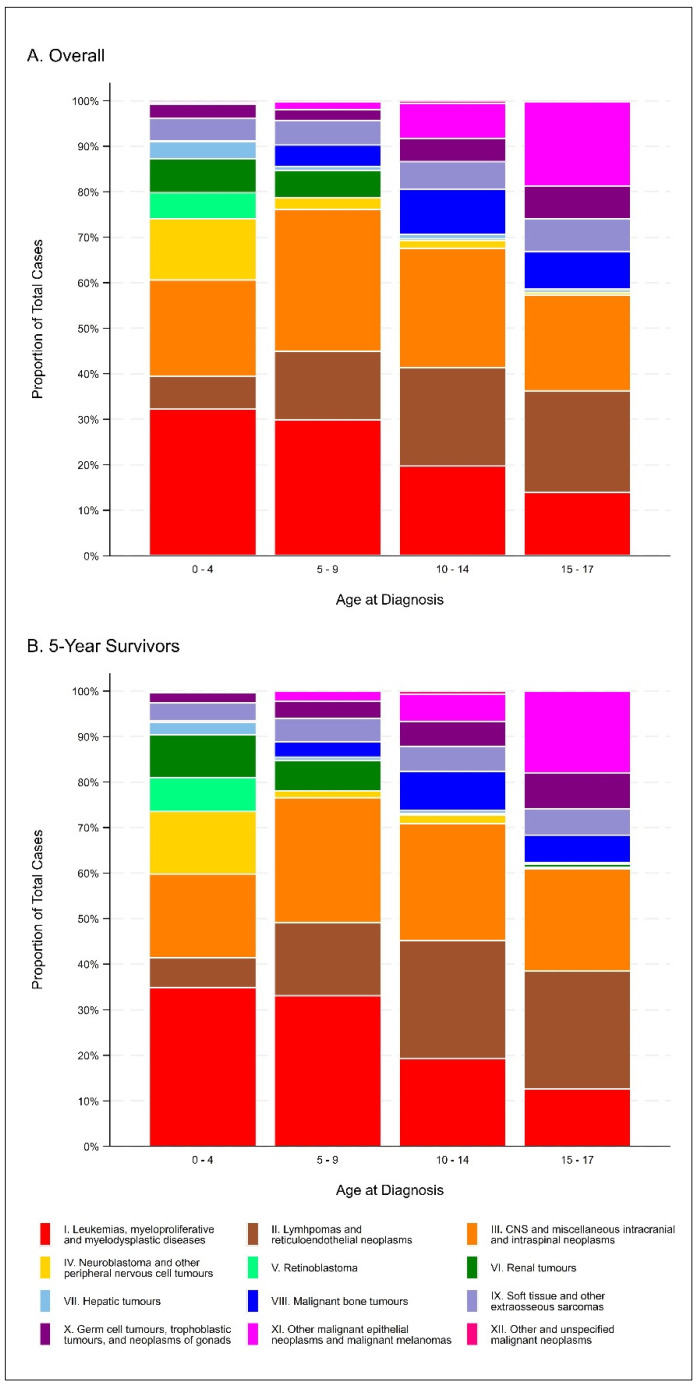
Distribution of ICCC-3 diagnosis categories, overall (**A**) and among 5-year survivors (**B**).

The corrected Table 2 appears below:

**Table 2:** Available chemotherapy treatment agents and radiotherapy with cumulative doses.

Chemotherapy Agent	Number of Survivors	Dose Available (%)	Median (mg/m^2^) (IQR)
Bleomycin Blenoxane Bleo	96	95 (99.0)	60.6 (46.0–69.1)
Busulphan Busulfan (Myleran)	4	4 (100.0)	346.3 (295.3–429.1)
Carboplatin CBDCA Paraplatin Carboplatinum	209	164 (78.5)	1480.5 (983.0–2250.2)
Carmustine (BCNU) Bis-Chloroethyl-Nitrosourea BiCNU	2	2 (100.0)	309.9 (58.4–561.4)
Cisplatin CDDP Platinol Cisplatinum Cis-diamminedicloro-platinum II P	232	232 (100.0)	368.4 (230.4–454.3)
Cyclophosphamide Cytoxan CTX Procytox	837	831 (99.3)	3002.6 (1124.9–5293.9)
Cytarabine (IT ONLY) Ara-C Cytosar Cytosine arabinoside	558	441 (79.0)	95.6 (75.4–188.3)
Cytarabine (ONLY IV ≥ 500 mg/m^2^ per dose) Ara-C Cytosar Cytosine arabinoside	146	145 (99.3)	15,009.4 (10,349.3–24,129.0)
Daunomycin Daunorubicin Cerubidine DNR	300	299 (99.7)	102.1 (96.5–166.2)
Doxorubicin Adriamycin ADR	786	780 (99.2)	109.9 (74.7–217.8)
Doxorubicin-Pegylated Liposomal (DOXIL) PLD	4	4 (100.0)	111.9 (78.0–436.7)
Etoposide (VP16) VePesid ETOP	564	559 (99.1)	1335.1 (598.9–1875.0)
Etoposide Phosphate	3	2 (66.7)	2107.5 (1200.0–3015.0)
Hydrocortisone (IT ONLY)	113	89 (78.8)	80.3 (40.8–162.6)
Idarubicin Idamycin 4-Demethoxydaunorubicin	28	28 (100.0)	11.0 (09.9–20.9)
Ifosfamide Isophosphamide IFOS Ifex Holoxan	217	215 (99.1)	20,354.5 (6052.1–46,236.1)
Lomustine (CCNU) CeeNU Chloroethyl-Cyclohexyl-Nitrosurea	20	18 (90.0)	445.8 (299.3–568.5)
Melphalan L-PAM Alkeran L-Sarcolysin	5	5 (100.0)	177.5 (100.4–192.5)
Methotrexate (IT ONLY) MTX Amethopterin	558	446 (79.9)	243.6 (113.1–300.0)
Methotrexate (IV ≥ 500 mg/m^2^ ONLY) MTX Amethopterin	355	352 (99.2)	14,491.6 (6319.5–20,000.0)
Mitoxantrone Novantrone DHAD Dihydrochloride	57	57 (100.0)	46.6 (34.4–48.5)
Oxaliplatin Eloxatin	4	4 (100.0)	178.6 (53.5–286.2)
Procarbazine PCB Natulan Matulane	7	6 (85.7)	733.0 (62.3–1600.0)
Teniposide (Vumon) VM-26	21	21 (100.0)	404.2 (383.7–529.0)
Thiotepa TESPA Triethylene Thiophosphoramide	12	11 (91.7)	502.2 (16.8–1271.8)
**Radiotherapy Site**	**Patients**	**Patients** **with** **Dosage** **(%)**	**Median (cGy)** **(IQR)**
All Sites Combined	525	513 (97.7)	2550 (1800–4776)
Abdomen	86	86 (100.0)	1490 (1080–2400)
Central Nervous System ^1^	266	265 (99.6)	3600 (1800–5400)
Chest	6	6 (100.0)	2325 (1500–4500)
Face ^2^	16	16 (100.0)	3870 (2000–4320)
Limb	43	42 (97.7)	4500 (2200–5000)
Liver	3	3 (100.0)	600 (450–1200)
Lung	32	32 (100.0)	1500 (1200–2130)
Lymph Nodes ^3^	56	56 (100.0)	2100.0 (2100–3075)
Nasopharynx	5	5 (100.0)	4150 (3600–4500)
Neck	56	56 (100.0)	2100 (2100–3375)
Pelvis	35	35 (100.0)	2100 (1050–3600)
Skull	18	17 (94.4)	2400 (1500–4500)
Spleen	11	11 (100.0)	2100 (2100–2100)
Testis	3	3 (100.0)	3600 (1200–8659)
Thorax ^4^	40	40 (100.0)	2100 (2100–2550)
Other	85	82 (96.5)	2603 (2000–5000)
Missing ^5^	17	3 (17.6)	2160 (1440–5400)

^1^ Category consists of site codes “Brain,” “Spine”, and “Craniospinal”. ^2^ Category consists of site codes “Face”, “Orbit”, and “Parotid”. ^3^ Category consists of site codes “Lymph Nodes” and “Mantle Nodes”. ^4^ Category consists of site codes “Mediastinum” and “Thorax”. ^5^ Category consists of site code “Not available” and records with missing site code value. Presented data are based on treatment information available as of 4 May 2022 (for Alberta Children’s Hospital) and 30 November 2022 (for Stollery Children’s Hospital).

The corrected Table 3 appears below:

**Table 3:** Characteristics of cohort participants who are not in the Cancer in Young People in Canada consortium.

Characteristic	Overall Number (%)	5-Year Survivors Number (%)
Total	836 (100.0)	482 (100.0)
Sex		
Male	399 (47.7)	238 (49.4)
Female	437 (52.3)	244 (50.6)
Time Since Diagnosis (years) ^1^		
0–<5	354 (42.3)	0 (0.0)
5–<10	243 (29.1)	243 (50.4)
10–<14	174 (20.8)	174 (36.1)
≥15	65 (7.8)	65 (13.5)
Median (IQR)	6.0 (2.2–10.8)	9.9 (7.1–13.2)
ICCC-3 Diagnosis Category		
Leukemias, myeloproliferative diseases, and myelodysplastic diseases	108 (12.9)	53 (11.0)
Lymphomas and reticuloendothelial neoplasms	144 (17.2)	99 (20.5)
CNS and miscellaneous intracranial and intraspinal neoplasms	247 (29.6)	147 (30.5)
Neuroblastoma and other peripheral nervous cell tumors	16 (1.9)	7 (1.5)
Retinoblastoma	40 (4.8)	31 (6.4)
Renal tumors	6 (0.7)	5 (1.0)
Hepatic tumors	3 (0.4)	1 (0.2)
Malignant bone tumors	45 (5.4)	19 (3.9)
Soft tissue and other extraosseous sarcomas	39 (4.7)	21 (4.4)
Germ cell tumors, trophoblastic tumors, and neoplasms of gonads	55 (6.6)	31 (6.4)
Other malignant epithelial neoplasms and malignant melanomas	131 (15.7)	67 (13.9)
Other and unspecified malignant neoplasms	2 (0.2)	1 (0.2)
Year of Diagnosis		
2001–2005	215 (25.7)	165 (34.2)
2006–2010	218 (26.1)	179 (37.1)
2011–2015	276 (33.0)	138 (28.6)
2016–2018	127 (15.2)	0 (0.0)
Age at Diagnosis (years)		
0–4	161 (19.3)	80 (16.6)
5–9	65 (7.8)	32 (6.6)
10–14	128 (15.3)	78 (16.2)
15–17	482 (57.7)	292 (60.6)
Median (IQR)	15.4 (8.5–16.8)	15.6 (11.0–17.0)
Attained Age (years) ^1^		
0–4	63 (7.5)	0 (0.0)
5–9	73 (8.7)	31 (6.4)
10–14	75 (9.0)	37 (7.7)
15–19	211 (25.2)	48 (10.0)
20–24	201 (24.0)	153 (31.7)
25–29	138 (16.5)	138 (28.6)
30–34	74 (8.9)	74 (15.4)
≥35	1 (0.1)	1 (0.2)
Median (IQR)	19.9 (14.8–25.2)	24.2 (20.4–28.2)
Zone of Diagnosis		
South	64 (7.7)	38 (7.9)
Calgary	285 (34.1)	151 (31.3)
Central	118 (14.1)	77 (16.0)
Edmonton	250 (29.9)	152 (31.5)
North	118 (14.1)	63 (13.1)
Alberta, zone unknown	1 (0.1)	1 (0.2)

^1^ Censored to date of death, last known date (i.e., loss to follow-up), or end of study (i.e., 31 December 2018), whichever occurs first.

The corrected Figure 2 appears below:

**Figure 2:**
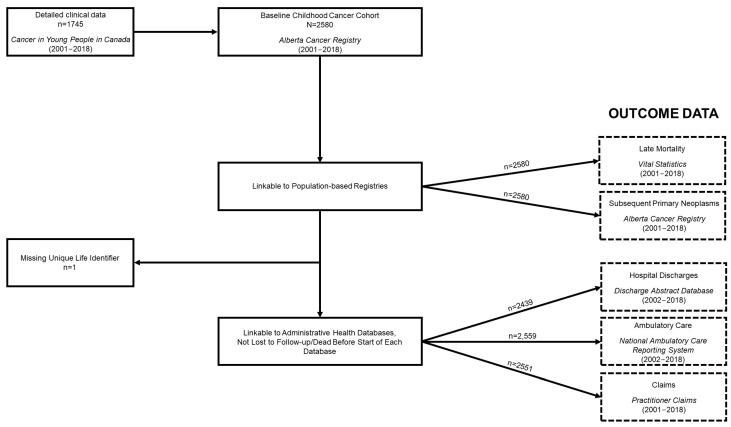
Alberta Childhood Cancer Survivorship Research Program cohort constitution flowchart.

The corrected Table 4 appears below:

**Table 4:** Late effects data, overall and after 5-year survivorship.

Database and Outcome	Overall (%)	5-Year Survivors (%)
Alberta Cancer Registry		
Subsequent primary neoplasms observed	94	55
Number of survivors	83 (3.2)	48 (3.5)
Vital Statistics		
Alive	2173 (84.2)	1347 (97.3)
Deceased	408 (15.8)	38 (2.7)
Discharge Abstract Database		
Number of inpatient events	16,669	995
Number of survivors	2254 (87.3)	331 (23.9)
National Ambulatory Care Reporting System		
Number of ambulatory/outpatient events	445,150	60,906
Number of survivors	2533 (98.1)	1296 (93.6)
Practitioner Claims		
Number of claims records	396,074	88,885
Number of survivors	2549 (98.8)	1317 (95.1)

**Supplementary Materials**

Due to inaccuracies in Table 3, the Supplementary Table S1 also needed to be updated. The corrected Supplementary Table S1 appears below:

**Table S1:** All chemotherapy agents available for 1379 survivors of childhood cancer.

Chemotherapy Agent	Number of Survivors	%
Anti-rejection drugs (Sirolimus Tacrolimus MMF)	13	0.1
Antithymocyte globulin [ATG/ATGAM]	2	<0.1
Arsenic trioxide (Trisinox)	4	<0.1
Asparaginase E-Coli (L-Asp) Elspar Kidrolase	126	1.2
Asparaginase Erwinia (Erwinase)	56	0.5
Asparaginase Peg	355	3.4
Azacytidine (Aza-C) 5-AZA 5-AC 5-azacytidine)	2	<0.1
Bevacizumab (Avastin)	4	<0.1
Bleomycin Blenoxane Bleo *	96	0.9
Blinatumomab	1	<0.1
Bortezomib (Velcade)	2	<0.1
Brentuximab vedotin (SGN-35)	5	0.1
Busulphan Busulfan (Myleran) *	4	<0.1
Carboplatin CBDCA Paraplatin Carboplatinum	209	2
Carmustine (BCNU) Bis-Chloroethyl-Nitrosourea BiCNU *	2	<0.1
Ch14.18 (Dinutuximab)	13	0.1
Cisplatin CDDP Platinol Cisplatinum Cis-diamminedicloro-platinum II P *	232	2.2
Cladribine CdA Leustatin	4	<0.1
Clofarabine Clolar	13	0.1
Colony stimulating factors/Erythropoietin (e.g., G-CSF Eprex Aransep)	384	3.7
Crizotinib	2	<0.1
Cyclophosphamide Cytoxan CTX Procytox *	837	8.1
Cyclosporin	55	0.5
Cytarabine (IT ONLY) Ara-C Cytosar Cytosine arabinoside	558	5.4
Cytarabine (IM sub q PO OR IV) Ara-C Cytosar Cytosine arabinoside	437	4.2
Cytarabine (ONLY IV >=500mg/m^2^ per dose) Ara-C Cytosar Cytosine arabinoside	146	1.4
Dabrafenib	2	<0.1
Dactinomycin (DACT) Actinomycin D Cosmogen Act-D	154	1.5
Dasatinib (BMS-354825)	6	0.1
Daunomycin Daunorubicin Cerubidine DNR *	300	2.9
Dexamethasone (Decadron)	474	4.6
Dexrazoxane Zinecard Cardioxane	24	0.2
Docetaxel (Taxotere)	4	<0.1
Dolastatin 10 (D10)	1	<0.1
Doxorubicin Adriamycin ADR *	786	7.6
Doxorubicin-pegylated liposomal (DOXIL) PLD *	4	<0.1
Erlotinib Tarceva OSI-774	3	<0.1
Etoposide (VP16) VePesid ETOP *	564	5.4
Etoposide phosphate	3	<0.1
Fludarabine FAMP Fludara	30	0.3
Fluorouracil (5-FU Adrucil Efudex Fluoroplex 5-fluorouracil)	35	0.3
Gamma globulin	68	0.7
Gemcitabine (Gemzar)	12	0.1
Gemtuzumab (Mylotarg)	6	0.1
Hu14.18-IL2	2	<0.1
Hydrocortisone (IT ONLY)	113	1.1
Hydroxyurea Hydroxycarbamide Hydrea	6	0.1
Idarubicin Idamycin 4-Demethoxydaunorubicin *	28	0.3
Ifosfamide Isophosphamide IFOS Ifex Holoxan *	217	2.1
Imatinib (Gleevec) IMAT	11	0.1
Inotuzumab	1	<0.1
Interferon	2	<0.1
Interleukin-2	11	0.1
Irinotecan (CPT-11) Camptosar	52	0.5
Isotretinoin 13-cis-Retinoic Acid	44	0.4
Ixabepilone	1	<0.1
Lestaurtinib (CEP-701)	1	<0.1
Lomustine (CCNU) CeeNU Chloroethyl-Cyclohexyl-Nitrosurea *	20	0.2
Melphalan L-PAM Alkeran L-sarcolysin *	5	0.1
Mercaptopurine (6-MP Purinethol 6-mercaptopurine	433	4.2
Methotrexate (IM PO Sub q IC OR IV < 500 mg/m^2^) MTX amethopterin	428	4.1
Methotrexate (IT ONLY) MTX amethopterin	558	5.4
Methotrexate (IV >= 500 mg/m^2^ ONLY) MTX amethopterin *	355	3.4
Mitotane Lysodren	2	<0.1
Mitoxantrone Novantrone DHAD Dihydrochloride *	57	0.6
Nelarabine (Arranon AraG)	13	0.1
Nimotuzumab	2	<0.1
Other	28	0.3
Oxaliplatin Eloxatin	4	<0.1
Paclitaxel Taxol	4	<0.1
Pembrolizumab	1	<0.1
Prednisone (Methylprednisone Prednisolone)	378	3.6
Procarbazine PCB Natulan Matulane *	7	0.1
Rituximab Rituxan	25	0.2
Sorafenib BAY 43-9006 Nexavar	8	0.1
Tamoxifen Tam Nolvadex	2	<0.1
Temozolomide TMZ Temodal	52	0.5
Teniposide (Vumon) VM-26 *	21	0.2
Thioguanine (6-TG Lanvis 6-thioguanine)	305	2.9
Thiotepa TESPA Triethylene Thiophosphoramide *	12	0.1
Topotecan (Hycamtin)	60	0.6
Trametinib	2	<0.1
Tretinoin ATRA all-trans-Retinoic acid Vesanoid	8	0.1
Vinblastine Velbe Velban VLB	57	0.6
Vincristine Leurocristine Oncovin VCR	976	9.4
Vinorelbine Navelbine	20	0.2
Vorinostat	1	<0.1

* Presented data based on treatment information available as of 4 May 2022 (for Alberta Children’s Hospital) and 30 November 2022 (for Stollery Children’s Hospital).

Additionally, we now supply Supplementary Table S2 which provides the numbers underlying Figure 2. Supplementary Table S2 appears below:

**Table S2:** Distribution of ICCC-3 diagnosis categories, overall and among 5-year survivors.

**ICCC-3 Diagnosis Category**	**Age at Diagnosis (Overall)**					**Age at Diagnosis (5-Year Survivors)**				
	**0–4**	**5–9**	**10–14**	**15–17**	**Total**	**0–4**	**5–9**	**1–14**	**15–17**	**Total**
**Leukemias, myeloproliferative diseases,** **and myelodysplastic diseases**	322 32.2	150 29.8	109 19.7	73 13.9	654 95.6	178 34.9	89 33.1	60 19.2	37 12.6	364 99.8
**Lymphomas and** **reticuloendothelial neoplasms**	72 7.2	76 15.1	120 21.7	117 22.3	385 66.3	33 6.5	43 16.0	81 26.0	76 25.9	233 74.3
**CNS and miscellaneous intracranial** **and intraspinal neoplasms**	212 21.2	157 31.2	145 26.2	110 21.0	624 99.6	94 18.4	74 27.5	80 25.6	66 22.4	314 94.0
**Neuroblastoma and other peripheral** **nervous cell tumors**	135 13.5	13 2.6	10 1.8	3 0.6	161 18.5	70 13.7	4 1.5	6 1.9	1 0.3	81 17.5
**Retinoblastoma**	57 5.7	0 0.0	0 0.0	0 0.0	57 5.7	38 7.5	0 0.0	0 0.0	0 0.0	38 7.5
**Renal tumors**	75 7.5	30 6.0	2 0.4	2 0.4	109 14.2	48 9.4	18 6.7	1 0.3	2 0.7	69 17.1
**Hepatic tumors**	37 3.7	4 0.8	5 0.9	2 0.4	48 5.8	14 2.7	2 0.7	2 0.6	1 0.3	19 4.5
**Malignant bone tumors**	2 0.2	24 4.8	55 9.9	43 8.2	124 23.1	2 0.4	9 3.3	27 8.7	18 6.1	56 18.5
**Soft tissue and other** **extraosseous sarcomas**	49 4.9	27 5.4	34 6.1	38 7.3	148 23.7	20 3.9	14 5.2	17 5.4	17 5.8	68 20.4
**Germ cell tumors, trophoblastic** **tumors, and neoplasms of gonads**	31 3.1	12 2.4	28 5.1	38 7.3	109 17.8	11 2.2	10 3.7	17 5.4	23 7.8	61 19.1
**Other malignant epithelial neoplasms** **and malignant melanomas**	4 0.4	9 1.8	43 7.8	97 18.5	153 28.5	0 0.0	6 2.2	19 6.1	53 18.0	78 26.3
**Other and unspecified** **malignant neoplasms**	4 0.4	1 0.2	3 0.5	1 0.2	9 1.3	2 0.4	0 0.0	2 0.6	0 0.0	4 1.0
**Total**	1000	503	554	524	2581	510	269	312	294	1385

Reported data: frequency (top); column percentage (bottom).

Supplementary Table S2 citation is added in the back matter of the manuscript:

**Supplementary Materials**: The following supporting information can be downloaded at: https://www.mdpi.com/article/10.3390/cancers15153932/s1, Table S1: All chemotherapy agents available for 1379 survivors of childhood cancer. Table S2: Distribution of ICCC-3 diagnosis categories, overall and among 5-year survivors.

The authors apologize for any inconvenience caused and state that the scientific conclusions are unaffected. This correction was approved by the Academic Editor. The original publication has also been updated.
