# Supplementary material for: Alberta Childhood Cancer Survivorship Research Program
Source: Cancers (Basel). 2023 Aug 2;15(15):3932. doi: 10.3390/cancers15153932 (PMC10417797; doi:10.3390/cancers15153932)
Supplement: Supplementary file 1 [file cancers-15-03932-s001.zip › cancers-2410569-supplementary.pdf]

Table S1. All chemotherapy agents available for 1379 survivors of childhood cancer.

**Chemotherapy Agent**	**Number of Survivors**	**%**
Anti-rejection drugs (Sirolimus Tacrolimus MMF)	13	0.1
Antithymocyte globulin[ATG/ATGAM]	2	<0.1
Arsenic trioxide (Trisinox)	4	<0.1
Asparaginase E-Coli (L-Asp) Elspar Kidrolase	126	1.2
Asparaginase Erwinia (Erwinase)	56	0.5
Asparaginase Peg	355	3.4
Azacytidine (Aza-C) 5-AZA 5-AC 5-azacytidine)	2	<0.1
Bevacizumab (Avastin)	4	<0.1
Bleomycin Blenoxane Bleo *	96	0.9
Blinatumomab	1	<0.1
Bortezomib (Velcade)	2	<0.1
Brentuximab vedotin (SGN-35)	5	0.1
Busulphan Busulfan (Myleran) *	4	<0.1
Carboplatin CBDCA Paraplatin Carboplatinum	209	2
Carmustine (BCNU) Bis-Chloroethyl-Nitrosourea BiCNU *	2	<0.1
Ch14.18 (Dinutuximab)	13	0.1
Cisplatin CDDP Platinol Cisplatinum Cis-diamminedicloro-platinum II P *	232	2.2
Cladribine CdA Leustatin	4	<0.1
Clofarabine Clolar	13	0.1
Colony stimulating factors/Erythropoietin (e.g. G-CSF Eprex Aransep)	384	3.7
Crizotinib	2	<0.1
Cyclophosphamide Cytoxan CTX Procytox *	837	8.1
Cyclosporin	55	0.5
Cytarabine (IT ONLY) Ara-C Cytosar Cytosine arabinoside	558	5.4
Cytarabine (IM sub q PO OR IV) Ara-C Cytosar Cytosine arabinoside	437	4.2
Cytarabine (ONLY IV >=500 mg/m^2 per dose) Ara-C Cytosar Cytosine arabinoside	146	1.4
Dabrafenib	2	<0.1
Dactinomycin (DACT) Actinomycin D Cosmogen Act-D	154	1.5
Dasatinib (BMS-354825)	6	0.1
Daunomycin Daunorubicin Cerubidine DNR *	300	2.9
Dexamethasone (Decadron)	474	4.6
Dexrazoxane Zinecard Cardioxane	24	0.2
Docetaxel (Taxotere)	4	<0.1
Dolastatin 10 (D10)	1	<0.1
Doxorubicin Adriamycin ADR *	786	7.6
Doxorubicin-pegylated liposomal (DOXIL) PLD *	4	<0.1
Erlotinib Tarceva OSI-774	3	<0.1
Etoposide (VP16) VePesid ETOP *	564	5.4
Etoposide phosphate	3	<0.1
Fludarabine FAMP Fludara	30	0.3
Fluorouracil (5-FU Adrucil Efudex Fluoroplex 5-fluorouracil)	35	0.3
Gamma globulin	68	0.7
Gemcitabine (Gemzar)	12	0.1
Gemtuzumab (Mylotarg)	6	0.1
Hu14.18-IL2	2	<0.1
Hydrocortisone (IT ONLY)	113	1.1
Hydroxyurea Hydroxycarbamide Hydrea	6	0.1
Idarubicin Idamycin 4-Demethoxydaunorubicin *	28	0.3
Ifosfamide Isophosphamide IFOS Ifex Holoxan *	217	2.1
Imatinib (Gleevec) IMAT	11	0.1
Inotuzumab	1	<0.1
Interferon	2	<0.1
Interleukin-2	11	0.1

Chemotherapy Agent	Number of Survivors	%
Irinotecan (CPT-11) Camptosar	52	0.5
Isotretinoin 13-cis-Retinoic Acid	44	0.4
Ixabepilone	1	<0.1
Lestaurtinib (CEP-701)	1	<0.1
Lomustine (CCNU) CeeNU Chloroethyl-Cyclohexyl-Nitrosurea *	20	0.2
Melphalan L-PAM Alkeran L-sarcolysin *	5	0.1
Mercaptopurine (6-MP Purinethol 6-mercaptopurine	433	4.2
Methotrexate (IM PO Sub q IC OR IV<500 mg/m^2) MTX amethopterin	428	4.1
Methotrexate (IT ONLY) MTX amethopterin	558	5.4
Methotrexate (IV >=500 mg/m^2 ONLY) MTX amethopterin *	355	3.4
Mitotane Lysodren	2	<0.1
Mitoxantrone Novantrone DHAD Dihydrochloride *	57	0.6
Nelarabine (Arranon AraG)	13	0.1
Nimotuzumab	2	<0.1
Other	28	0.3
Oxaliplatin Eloxatin	4	<0.1
Paclitaxel Taxol	4	<0.1
Pembrolizumab	1	<0.1
Prednisone (Methylprednisone Prednisolone)	378	3.6
Procarbazine PCB Natulan Matulane *	7	0.1
Rituximab Rituxan	25	0.2
Sorafenib BAY 43-9006 Nexavar	8	0.1
Tamoxifen Tam Nolvadex	2	<0.1
Temozolomide TMZ Temodal	52	0.5
Teniposide (Vumon) VM-26 *	21	0.2
Thioguanine (6-TG Lanvis 6-thioguanine)	305	2.9
Thiotepa TESPA Triethylene Thiophosphoramide *	12	0.1
Topotecan (Hycamtin)	60	0.6
Trametinib	2	<0.1
Tretinoin ATRA all-trans-Retinoic acid Vesanoid	8	0.1
Vinblastine Velbe Velban VLB	57	0.6
Vincristine Leurocristine Oncovin VCR	976	9.4
Vinorelbine Navelbine	20	0.2
Vorinostat	1	<0.1

* Presented data based on treatment information available as of 4 May 2022 (for Alberta Children's Hospital) and 30 November 2022 (for Stollery Children's Hospital).

Table S2. Distribution of ICCC-3 diagnosis categories, overall and among 5-year survivors.

ICCC-3 Diagnosis Category	Age at Diagnosis (Overall)					Age at Diagnosis (5-Year Survivors)				
	0–4	5–9	10–14	15–17	Total	0–4	5–9	1–14	15–17	Total
Leukemias, myeloproliferative diseases, and myelodysplastic diseases	322	150	109	73	654	178	89	60	37	364
	32.2	29.8	19.7	13.9	95.6	34.9	33.1	19.2	12.6	99.8
Lymphomas and reticuloendothelial neoplasms	72	76	120	117	385	33	43	81	76	233
	7.2	15.1	21.7	22.3	66.3	6.5	16.0	26.0	25.9	74.3
CNS and miscellaneous intracranial and intraspinal neoplasms	212	157	145	110	624	94	74	80	66	314
	21.2	31.2	26.2	21.0	99.6	18.4	27.5	25.6	22.4	94.0
Neuroblastoma and other peripheral nervous cell tumors	135	13	10	3	161	70	4	6	1	81
	13.5	2.6	1.8	0.6	18.5	13.7	1.5	1.9	0.3	17.5
Retinoblastoma	57	0	0	0	57	38	0	0	0	38
	5.7	0.0	0.0	0.0	5.7	7.5	0.0	0.0	0.0	7.5
Renal tumors	75	30	2	2	109	48	18	1	2	69
	7.5	6.0	0.4	0.4	14.2	9.4	6.7	0.3	0.7	17.1
Hepatic tumors	37	4	5	2	48	14	2	2	1	19
	3.7	0.8	0.9	0.4	5.8	2.7	0.7	0.6	0.3	4.5
Malignant bone tumors	2	24	55	43	124	2	9	27	18	56
	0.2	4.8	9.9	8.2	23.1	0.4	3.3	8.7	6.1	18.5
Soft tissue and other extraosseous sarcomas	49	27	34	38	148	20	14	17	17	68
	4.9	5.4	6.1	7.3	23.7	3.9	5.2	5.4	5.8	20.4
Germ cell tumors, trophoblastic tumors, and neoplasms of gonads	31	12	28	38	109	11	10	17	23	61
	3.1	2.4	5.1	7.3	17.8	2.2	3.7	5.4	7.8	19.1
Other malignant epithelial neoplasms and malignant melanomas	4	9	43	97	153	0	6	19	53	78
	0.4	1.8	7.8	18.5	28.5	0.0	2.2	6.1	18.0	26.3
Other and unspecified malignant neoplasms	4	1	3	1	9	2	0	2	0	4
	0.4	0.2	0.5	0.2	1.3	0.4	0.0	0.6	0.0	1.0
**Total**	1000	503	554	524	2,581	510	269	312	294	1385

Reported data: frequency (top); column percentage (bottom).
